# Whole-Genome Sequencing of a Chlorimuron-Ethyl-Degrading Strain: Chenggangzhangella methanolivorans CHL1 and Its Degrading Enzymes

**DOI:** 10.1128/spectrum.01822-22

**Published:** 2022-07-21

**Authors:** Zhixiong Yu, Wu Gu, Yi Yang, Xiang Li, Xinyu Li, Tingting Li, Jian Wang, Zhencheng Su, Xu Li, Yumeng Dai, Mingkai Xu, Huiwen Zhang

**Affiliations:** a Key Laboratory of Pollution Ecology and Environmental Engineering, Institute of Applied Ecology, Chinese Academy of Sciences, Shenyang, China; b University of Chinese Academy of Sciences, Beijing, China; c Shenyang Research Institute of Chemical Industry, Shenyang, China; Oklahoma State University

**Keywords:** gene knockout, gene complementation, microbial degradation, pollutant, sulfonylurea herbicide, whole-genome sequencing

## Abstract

Chlorimuron-ethyl is a commonly used sulfonylurea herbicide, and its long-term residues cause serious environmental problems. Biodegradation of chlorimuron-ethyl is effective and feasible, and many degrading strains have been obtained, but still, the genes and enzymes involved in this degradation are often unclear. In this study, whole-genome sequencing was performed on chlorimuron-ethyl-degrading strain, Chenggangzhangella methanolivorans CHL1. The complete genome of strain CHL1 contains one circular chromosome of 5,542,510 bp and a G+C content of 68.17 mol%. Three genes, *sulE*, *pnbA*, and *gst*, were predicted to be involved in the degradation of chlorimuron-ethyl, and this was confirmed by gene knockout and gene complementation experiments. The three genes were cloned and expressed in Escherichia coli BL21 (DE3) to allow for the evaluation of the catalytic activities of the respective enzymes. The glutathione-*S*-transferase (GST) catalyzes the cleavage of the sulfonylurea bridge of chlorimuron-ethyl, and the esterases, PnbA and SulE, both de-esterify it. This study identifies three key functional genes of strain CHL1 that are involved in the degradation of chlorimuron-ethyl and also provides new approaches by which to construct engineered bacteria for the bioremediation of environments polluted with sulfonylurea herbicides.

**IMPORTANCE** Chlorimuron-ethyl is a commonly used sulfonylurea herbicide, worldwide. However, its residues in soil and water have a potent toxicity toward sensitive crops and other organisms, such as microbes and aquatic algae, and this causes serious problems for the environment. Microbial degradation has been demonstrated to be a feasible and promising strategy by which to eliminate xenobiotics from the environment. Many chlorimuron-ethyl-degrading microorganisms have been reported, but few studies have investigated the genes and enzymes that are involved in the degradation. In this work, two esterase-encoding genes (*sulE*, *pnbA*) and a glutathione-*S*-transferase-encoding gene (*gst*) responsible for the detoxification of chlorimuron-ethyl by strain Chenggangzhangella methanolivorans CHL1 were identified, then cloned and expressed in Escherichia coli BL21 (DE3). These key chlorimuron-ethyl-degrading enzymes are candidates for the construction of engineered bacteria to degrade this pesticide and enrich the resources for bioremediating environments polluted with sulfonylurea herbicides.

## INTRODUCTION

Chlorimuron-ethyl, which is a sulfonylurea herbicide, is widely used to prevent the growth of broad-leaved weeds and sedge in soybean (1). However, the excessive application of chlorimuron-ethyl leads to long-term residue in soils. This can cause many ecological problems (2), such as soil degradation, toxic impact on susceptible crops, and effects on the soil microbial community (3–5). It is therefore necessary to eliminate chlorimuron-ethyl residues in soil. The degradation of chlorimuron-ethyl occurs mainly by biodegradation and chemical hydrolysis. Biodegradation strategies, which have high efficiency and are environmentally friendly, have been extensively studied (6–8).

In recent years, many chlorimuron-ethyl degrading microorganisms have been isolated, but their mechanisms of degradation have not been fully studied (9–11). Some studies have investigated the degrading enzymes and their coding genes (12). For example, two cytochrome P450 monooxygenases of Streptomyces griseolus and flavin-containing monooxygenases of Talaromyces flavus LZM1 broke the sulfonylurea bridge of sulfonylurea herbicides by oxidation (13–15). A hydrolase was obtained from Oceanisphaera psychrotolerans, which was expressed in Escherichia coli and hydrolyzed the sulfonylurea bridge (16). Esterase (SulE) from Hansschlegelia zhihuaiae S113 hydrolyzed the ester bond to degrade sulfonylurea herbicides (17). Carboxylesterase (CarE) of Rhodococcus erythropolis D310-1 de-esterified chlorimuron-ethyl (18). Glutathione-*S*-transferase (GST) from Klebsiella jilinsis 2N3 catalyzed the degradation of chlorimuron-ethyl (19). Two other key proteins (Kj-CysJ and Kj-SsuD) of K. jilinsis 2N3 were also involved in the degradation of chlorimuron-ethyl (20).

Strain Chenggangzhangella methylocysteaceae CHL1 is a novel sulfonylurea herbicide-degrading microorganism that was recently isolated and identified by our laboratory (8, 21). In the present study, the whole-genome of strain CHL1 was sequenced using Pacific Bioscience sequencing technology. On the basis of sequence and database annotations, genes involved in the degradation of chlorimuron-ethyl were predicted and subsequently confirmed by gene knockout and gene complementation experiments. The recombinant enzymes encoded by the key degradation genes were expressed in E. coli, and their catalytic activities were evaluated. The enzymatic degradation mechanisms of chlorimuron-ethyl were analyzed via ultra-high-performance liquid chromatography with Q Exactive high-resolution accurate mass spectrometry (UPLC-Q Exactive HRMS). This study lays a foundation for understanding the chlorimuron-ethyl degradation mechanism of strain CHL1 and also provides new approaches for the bioremediation of environments polluted with sulfonylurea herbicides.

## RESULTS

### Genome sequencing of strain CHL1.

The complete genome of strain CHL1 contained one circular chromosome that was 5,542,510 bp long, with a G+C content of 68.17 mol%. The percentage of the protein coding genes was 87.32%, with a G+C content of 68.60 mol%. The mean coding sequence was 710.61 bp long. A total of 55 tRNAs and 6 rRNAs were predicted.

### Key genes for chlorimuron-ethyl degradation and phylogenetic analyses.

Glutathione-S-transferase from K. jilinsis 2N3, esterase SulE from H. zhihuaiae S113, and carboxylesterase CarE from R. erythropolis D310-1, previously reported to degrade chlorimuron-ethyl, were used as baits for a Basic Local Alignment Search Tool (BLAST) against the strain CHL1 genome (17–19). A SulE sequence identical to the SulE from H. zhihuaiae strain S113 was identified by BLAST (Table S1) (17). SulE is an esterase that de-esterifies sulfonylurea herbicides to their corresponding parent acid forms (Table S1) (17). In addition, a carboxylesterase of strain CHL1, PnbA, with high homology to the carboxylic ester hydrolase of H. zhihuaiae (68.27% amino acid similarity), was also identified (Fig. S1a; Table S1). H. zhihuaiae was isolated from polluted farmland soil and is capable of degrading the sulfonylurea herbicide metsulfuron-methyl via its carboxylic ester hydrolase activity. Thus, the PnbA of strain CHL1 might be involved in the de-esterification of chlorimuron-ethyl (22, 23). In addition to SulE and PnbA, a GST homologue was annotated in the categories “Drug metabolism-cytochrome P450” and “Metabolism of xenobiotics by cytochrome P450” by the Kyoto Encyclopedia of Genes and Genomes (KEGG) database (Table S1). The GST of strain CHL1 has high homology to the GSTs from Methylobacterium dankookense and M. gnaphalii (Fig. S1b). The GST from K. jilinsis 2N3 can break the sulfonylurea bridge of chlorimuron-ethyl (19). Therefore, the GST of strain CHL1 was predicted to participate in the cleavage of the sulfonylurea bridge. We hypothesize that the genes *sulE*, *pnbA*, and *gst* are encoding chlorimuron-ethyl degradation enzymes (Table S1).

### Gene knockout, gene complementation, and degradation of chlorimuron-ethyl.

**FIG 1:**
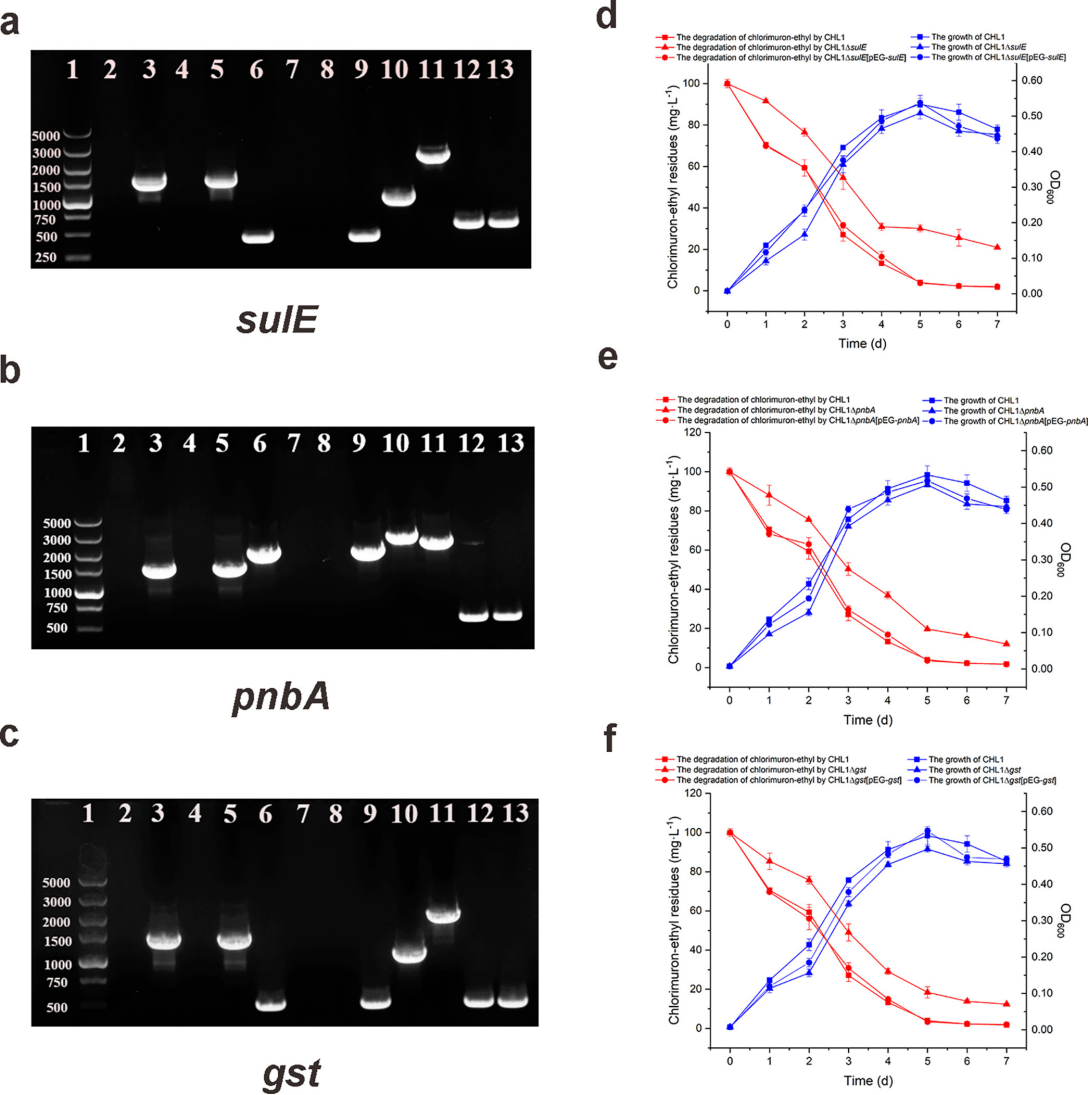
PCR verification and the degradation of chlorimuron-ethyl by mutant strains of strain CHL1 with *sulE* (a, d), *pnbA* (b, e), and *gst* (c, f) knockout and complementation. (a–c) Lane 1: DL_5000_ DNA marker. Lanes 2, 6, 10: wild-type strain CHL1. Lanes 3, 7, 11: knockout strain with kanamycin resistance gene. Lanes 4, 8, 12: knockout strain without kanamycin resistance gene. Lanes 5, 9, 13: complementation strain. Lanes 2, 3, 4, 5: amplification of the kanamycin resistance gene. Lanes 6, 7, 8, 9: amplification of the target gene using internal primers. Lanes 10, 11, 12, 13: amplification of the target gene, using external primers with 300 bp each, upstream and downstream. (d–f) Growth curves of strains (blue) and the degradation of chlorimuron-ethyl (red).

To provide experimental evidence to verify the function of chlorimuron-ethyl degradation enzymes, the coding genes of SulE, PnbA, and GST were knocked out by the Lambda-Red system. The kanamycin resistance gene cassette was inserted into the position of the target gene by homologous recombination technology, and the expression of the flanking genes around the target gene was not affected (24, 25). Construction of the mutant strains of strain CHL1 with *sulE*, *pnbA*, and *gst* knockout and complementation was confirmed by agarose gel electrophoresis (Fig. 1a–c). The growth and chlorimuron-ethyl degradation abilities of the knockout strains, the complemented strains, and the wild-type strain are shown in Fig. 1d–f. The biomasses of the knockout strains (CHL1Δ*sulE*, CHL1Δ*pnbA*, CHL1Δ*gst*) and the complemented strains (CHL1Δ*sulE*[pEG-*sulE*], CHL1Δ*pnbA*[pEG-*pnbA*], CHL1Δ*gst*[pEG-*gst*]) were similar to that of the wild-type on the seventh day. Compared to the wild-type strain, on the seventh day, the ability of the knockout strains, CHL1Δ*sulE*, CHL1Δ*pnbA*, and CHL1Δ*gst,* to degrade chlorimuron-ethyl decreased by 19.5%, 10.4%, and 10.8%, respectively (*P* < 0.05). The degradation ability of the complemented strains, CHL1Δ*sulE*[pEG-*sulE*], CHL1Δ*pnbA*[pEG-*pnbA*], and CHL1Δ*gst*[pEG-*gst*], was not significantly different from that of the wild-type (*P* > 0.05). These results show that *sulE*, *pnbA*, and *gst* directly or indirectly participate in the degradation of chlorimuron-ethyl by strain CHL1.

### Sequence analysis.

**FIG 2:**
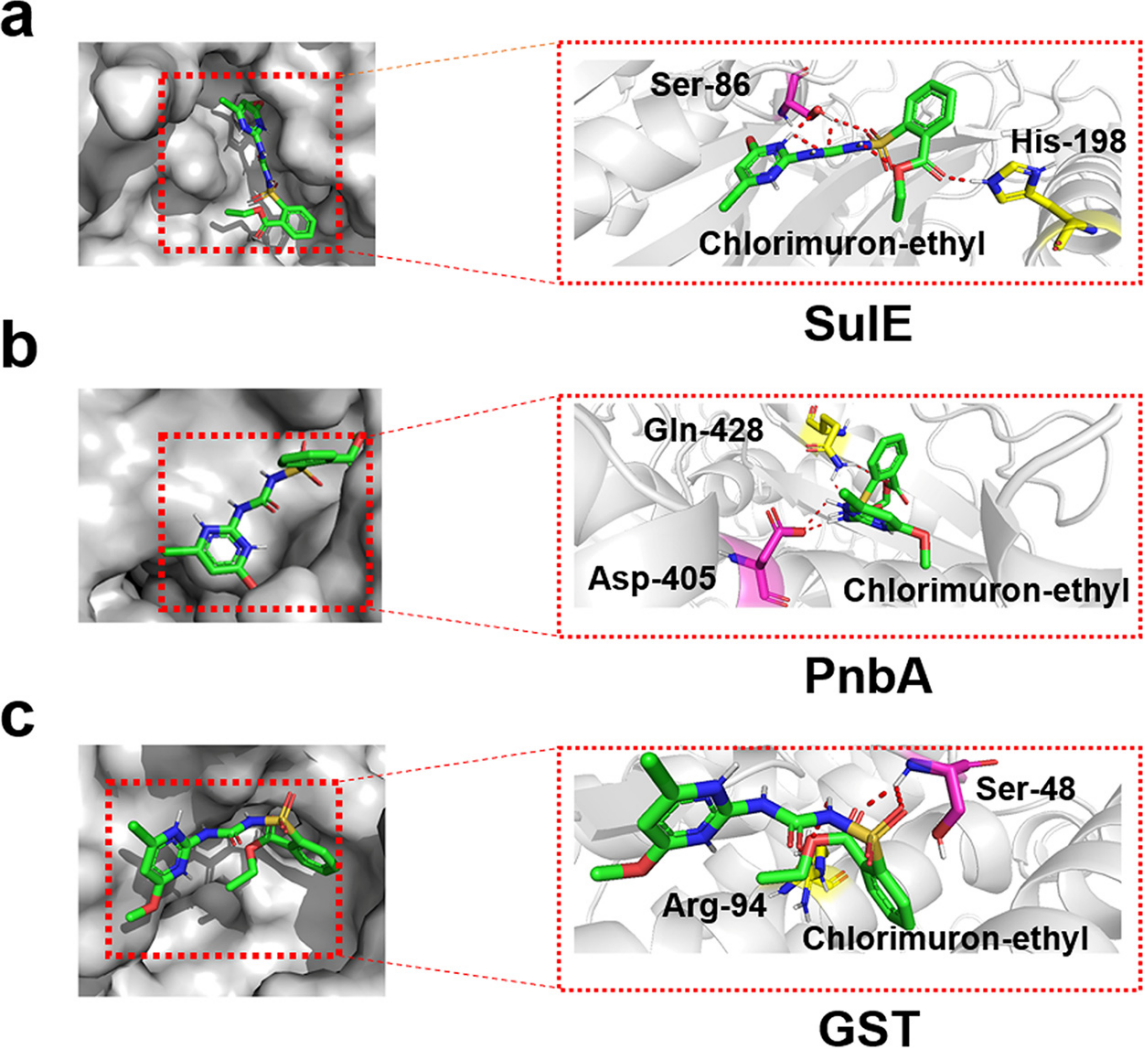
Binding site of SulE (a), PnbA (b), and GST (c) with chlorimuron-ethyl. (a) Ser-86 and His-198. (b) Asp405 and Gln-428. (c) Ser-48 and Arg-94.

The genes *sulE*, *pnbA*, and *gst* are located in different gene clusters (Fig. S2). SulE, PnbA, and GST were composed of 360 amino acids (1,083 bp), 594 amino acids (1,785 bp), and 203 amino acids (612 bp), respectively. SulE, PnbA, and GST were predicted to contain the structural domains, Esterase_713_like-3 (abhydrolase family), abhydrolase superfamily, and GST_C_7 (GST_C_7 family), respectively. The predicted binding sites for substrate chlorimuron-ethyl and SulE, PnbA, and GST were residues Ser-86 and His-198 (Fig. 2a), Ser-48 and Arg-94 (Fig. 2b), and Asp405 and Gln-428 (Fig. 2c), respectively.

### Heterologous expression.

**FIG 3:**
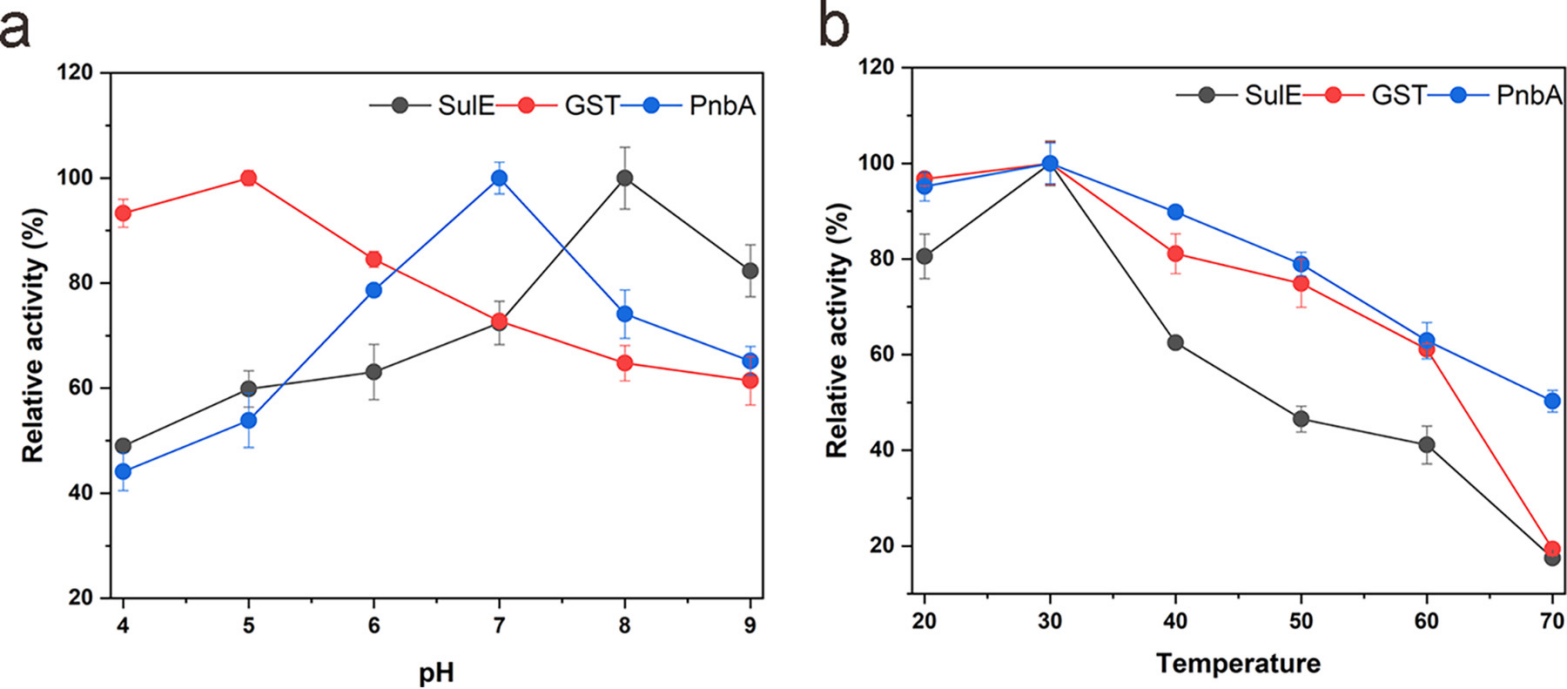
Effect of pH (a) and temperature (b) on the activity of SulE, PnbA, and GST toward chlorimuron-ethyl.

Through heterologous expression assay in vitro, the mechanisms of chlorimuron-ethyl degradation enzymes will be further revealed. GST, PnbA, and SulE of strain CHL1 were successfully expressed in E. coli BL21 (DE3) and purified by Ni-NTA agarose chromatography. The concentrations of the purified GST and PnbA enzymes were 207.93 μg · mL^−1^ and 256.98 μg · mL^−1^, respectively, and their activities in the degradation of chlorimuron-ethyl were 1.62 U · mL^−1^ and 0.98 U · mL^−1^, respectively. The optimal pH values for chlorimuron-ethyl degradation by GST and PnbA were 5.0 and 7.0, respectively, and 30°C was the optimum reaction temperature for both GST and PnbA (Fig. 3). The activity of purified SulE in our study (554.44 μg · mL^−1^) was consistent with that documented in previous reports (17, 26, 27).

### Enzymatic degradation mechanisms of chlorimuron-ethyl.

**FIG 4:**
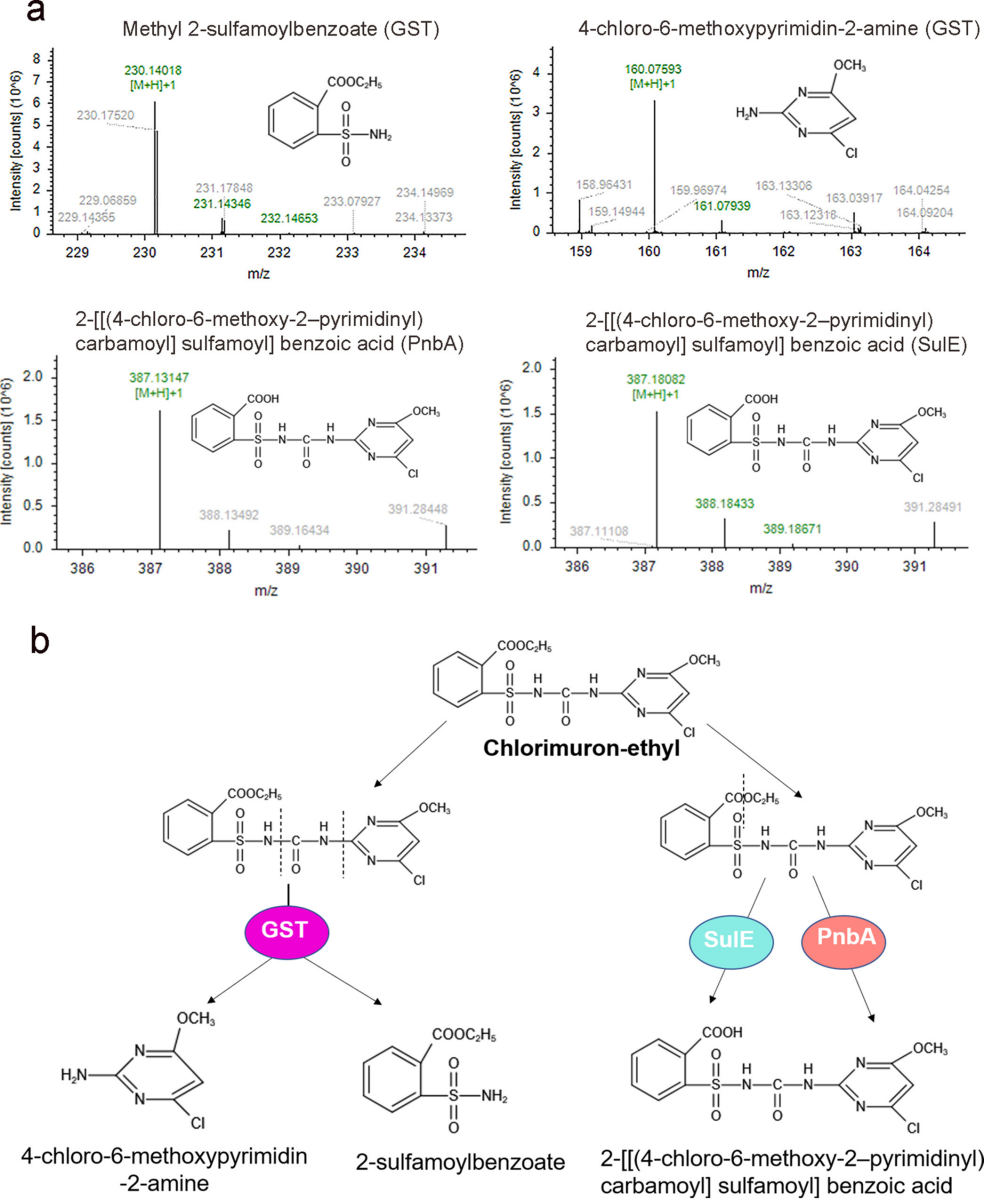
Degradation products (a) and degradation pathways (b) of chlorimuron-ethyl by SulE, PnbA, and GST.

The mass spectral ion peaks (*m/z*) of the chlorimuron-ethyl degradation products produced by GST, PnbA, and SulE from strain CHL1 were at 160.08 [M+H]^+^ and 230.14 [M+H]^+^, 387.13 [M+H]^+^, and 387.18 [M+H]^+^, respectively (Fig. 4a). On the basis of these results and the literature, the degradation products of chlorimuron-ethyl by GST were determined to be 4-chloro-6-methoxypyrimidin-2-amine and 2-sulfamoylbenzoate, and the degradation product of chlorimuron-ethyl by PnbA and SulE was 2-{[(4-chloro-6-methoxy-2-pyrimidinyl) carbamoyl] sulfamoyl} benzoic acid (17–19). Considering these products, the degradation mechanisms of chlorimuron-ethyl by the enzymes were analyzed. SulE and PnbA could de-esterify chlorimuron-ethyl to produce 2-{[(4-chloro-6-methoxy-2-pyrimidinyl) carbamoyl] sulfamoyl} benzoic acid, as reported previously for SulE (Fig. 4b) (17). GST catalyzed the cleavage of the sulfonylurea bridge of chlorimuron-ethyl to produce 2-sulfamoylbenzoate and 4-chloro-6-methoxypyrimidin-2-amine (Fig. 4b).

## DISCUSSION

High-efficiency, sulfonylurea herbicide-degrading strain CHL1 represents a novel genus and species of the family Methylocystaceae (28). Here, the whole-genome of strain CHL1 was sequenced and analyzed. The results have important theoretical and practical significance. The genes *sulE*, *pnbA*, and *gst* were predicted to be related to the degradation of chlorimuron-ethyl by strain CHL1, which was verified by gene knockout and gene complementation experiments (17–19, 22, 23). H. zhihuaiae strain S113 almost completely lost its ability to degrade chlorimuron-ethyl after *sulE* was knocked out (17). In comparison, strain CHL1Δ*sulE* retained the ability to degrade 79.1% of the chlorimuron-ethyl. Moreover, the chlorimuron-ethyl degradation rates of strains CHL1Δ*pnbA* and CHL1Δ*gst* were >80%. Some other genes or pathways may be a response for chlorimuron-ethyl degradation in strain CHL1, or this strain many contain multiple isoenzymes with the same or similar degradation functions (19).

The predicted substrate binding sites for chlorimuron-ethyl were residues Ser-86 and His-198 of SulE, Asp405, and Gln-428 of PnbA as well as Ser-48 and Arg-94 of GST. In the future, these key amino acid sites will be verified by site-directed mutagenesis and modified aiming to improve the enzyme activities (29). SulE, PnbA, and GST from strain CHL1 were successfully recombinantly expressed in E. coli and purified. The optimum temperature for the degradation of chlorimuron-ethyl by SulE, GST, and PnbA was 30°C in each case, and the optimal pH values for the reactions were 8.0, 5.0, and 7.0, respectively. Considering the mechanisms of degradation of chlorimuron-ethyl, GST cleaved the sulfonylurea bridge, and SulE and PnbA broke the ester bond. The results were consistent with the previous functional prediction for GST and PnbA in this experiment. GST degraded chlorimuron-ethyl to 2-sulfamoylbenzoate and 4-chloro-6-methoxypyrimidin-2-amine. The product 4-chloro-6-methoxypyrimidin-2-amine was previously identified in the degradation of chlorimuron-ethyl by Pseudomonas sp. LW3, R. erythropolis D310-1, and K. jilinsis 2N3 (6, 18, 19). Furthermore, 2-sulfamoylbenzoate was an intermediate of the degradation product *o*-sulfonate benzoic imide in chlorimuron-ethyl degradation by GST of K. jilinsis 2N3 (19). SulE and PnbA both degraded chlorimuron-ethyl to generate 2-{[(4-chloro-6-methoxy-2-pyrimidinyl) carbamoyl] sulfamoyl} benzoic acid, which was previously identified in the degradation of chlorimuron-ethyl by R. erythropolis D310-1 and H. zhihuaiae S113 (17, 18).

It is necessary to study the genes and enzymes involved in herbicide degradation to remediate herbicide-contaminated environments (30–32). The known genes and enzymes involved in the degradation of sulfonylurea herbicides are still limited, and they need to be explored further. Our results reveal the genome sequence of strain CHL1 and its key genes and enzymes for degrading chlorimuron-ethyl, which could provide candidates for the construction of engineered bacteria for degradation and could enrich the resources of sulfonylurea herbicide-degrading enzymes for environmental bioremediation.

## MATERIALS AND METHODS

### Plasmids, bacterial strains, chemicals, and media.

Chenggangzhangella methanolivorans strain CHL1 was isolated from sludge samples collected from sewage treatment tanks at an agricultural chemical factory (Jiangsu Province, China) through enrichment, and had been identified and stored in our laboratory (8). All of the plasmids and strains used in this study are displayed in Table 1.

**TABLE 1:** The plasmids and bacterial strains used in this study

Strains and plasmids	Relevant genotype or characteristic	Reference or source
Plasmid pKD4	Knockout vector, Kan^R^	This study
Plasmid pKD46	Knockout vector, Amp^R^	This study
Plasmid pCP20	Knockout vector, Amp^R^	This study
Plasmid pEarleyGate100	Complementation vector, Kan^R^	Huiyuanyuan Biotechnology
Plasmid pET-28a (+)	Expression vector, Kan^R^	This study
Plasmid pEG-*gst*	*Gst*, *pnbA*, and *sulE* complementation vectors by pEarleyGate100, respectively	
Plasmid pEG-*pnbA*		
Plasmid pEG-*sulE*		
Strain CHL1	Chlorimuron-ethyl utilizer, wild-type	This study
Strain CHL1Δ*gst*	Strain CHL1 with *gst*, *pnbA*, and *sulE* deleted, respectively	
Strain CHL1Δ*pnbA*		
Strain CHL1Δ*sulE*		
Strain CHL1Δ*gst*[pEG-*gst*]	*Gst*, *pnbA*, and *sulE* were complemented by pEG-*gst*, pEG-*pnbA*, and pEG-*sulE* plasmids in CHL1Δ*gst*, CHL1Δ*pnbA*, and CHL1Δ*sulE* strains, respectively	
Strain CHL1Δ*pnbA*[pEG-*pnbA*]		
Strain CHL1Δ*sulE*[pEG-*sulE*]		
Strain CHL1[pKD46]	Strain CHL1 with plasmid pKD46	This study
E. coli BL21 (DE3)	F_ompT hsdS (rB_mB_) dcm gal (DE3)	Solarbio Biotechnology

The chlorimuron-ethyl and the reagents for the spectroscopic and chromatographic analyses were purchased from Sigma-Aldrich (Shanghai, China). All chemicals and analytical reagents were provided by TaKaRa Biotechnology (Dalian, China). The components of the mineral salt medium (MSM) were as follows: 2.0 g NaNO_3_, 0.5 g NaCl, 2.0 g KH_2_PO_4_, 0.02 g FeSO_4_·7H_2_O, 0.125 g MgSO_4_·7H_2_O, 0.2 g yeast extract, and 20 mL methanol per 1,000 mL of distilled water, pH 7.0.

### Genome sequencing, annotation, and analysis of strain CHL1.

Strain CHL1 was cultured on MSM solid from a freeze-dried stock and incubated at 28°C. A single colony was selected and cultured in MSM to the exponential phase (160 rpm, 28°C). The culture medium was centrifuged (8,000 rpm, 5 min), and the harvested cells were washed repeatedly with PBS buffer (0.2 mol·L^−1^, pH 7.8) and suspended in MSM (8). The bacterial precipitations were collected by centrifugation (8,000 rpm, 5 min) to extract the genomic DNA of strain CHL1 using a bacterial genomic DNA extraction kit (Tiangen, Beijing).

Sequencing of the genomic DNA of strain CHL1 was performed by Personal Biotechnology Co. (Guangzhou, China), using Pacific Bioscience Sequel technology (33–35). The complete genome sequence of strain CHL1 was assembled using Mecat2 and Unicycle and was annotated using the Non-Redundant Protein Sequence (NR), Swiss-Prot Protein Sequence (Swiss-Prot), Pfam protein domain (Pfam), Clusters of Orthologous Groups of Proteins (COG), Gene Ontology (GO), and Kyoto Encyclopedia of Genes and Genomes (KEGG) databases. Key genes involved in the degradation of chlorimuron-ethyl by strain CHL1 were predicted based on the genome sequence combined with literature about degradation enzymes for sulfonylurea herbicides.

### Phylogenetic analyses.

On the basis of the protein database UniProt and sequence alignment, the sequences with the top 10 similarities were selected for construction of phylogenetic trees in MEGA 7.0 software (36), using the neighbor-joining method and 1,000 bootstrap replicates.

### Gene knockout and complementation.

Lambda-Red recombination technology was used as the gene replacement method, based on the recombination of functional genes, *exo*, *bet*, and *gam*, of the Lambda phage (37). The Lambda-Red system is widely used to verify gene function by replacing a target gene with an antibiotic resistance gene to achieve mutation (20, 38). *sulE*, *gst*, and *pnbA* were knocked out from strain CHL1 using the Lambda-Red system to test the roles of the respective gene products in degrading chlorimuron-ethyl (39). The primers used in the experiments are listed in Table S2. Strain CHL1 was cultured in 100 mL of MSM at 28°C with shaking at 160 rpm until the logarithmic growth phase (OD_600 nm_ = 0.8). The bacterial suspension was centrifuged at 9,500 rpm for 10 min and washed with pre-cooled 10% glycerol (4°C) four times to obtain competent cells.

The plasmid pKD46 was transformed into competent cells of strain CHL1 using a Gene Pulser Xcell (Bio-Rad) with parameters, capacitance 25 μF, voltage 1,800 V, resistance 200 Ω, and pulse time 5 ms. Competent cells of strain CHL1[pKD46] were prepared as described above, and the expression of the recombinases was induced by the addition of 100 mM arabinose.

Plasmid pKD4 contained the kanamycin resistance gene, and the 5′-end of each of the two primers contained a sequence of 60 bp, homologous to either end of *gst*, *pnbA*, or *sulE*, respectively. The gene-targeting linear box was amplified by one-step PCR, using the primers with homologous arms, pKD4, and PrimeSTAR HS DNA polymerase (TaKaRa) (Fig. S3a). Then, to obtain the knockout strains, it was transformed into competent cells of strain CHL1[pKD46] using the Gene Pulser Xcell with the same parameters as described above.

Plasmid pCP20, containing two FLP recombination target sites, was transformed into competent cells of the knockout strains using the Gene Pulser Xcell with the same parameters as described above to remove the kanamycin resistance gene (Fig. S3b).

Plasmid pEarleyGate100 was used to construct the corresponding complementation strains. *sulE* and *gst* amplified by PCR were ligated into pEarleyGate100, using the XbaI and HindIII restriction sites, respectively (Fig. S4). *pnbA* amplified by PCR was ligated into pEarleyGate100 using the XbaI and SpeI restriction sites. The resulting plasmids, pEG-*gst*, pEG-*pnbA*, and pEG-*sulE*, were transformed into the corresponding knockout strain to obtain the complemented strain.

The construction of mutants was confirmed by PCR of (i) the kanamycin resistance gene, (ii) the target gene, and (iii) the full length of the target gene (with 300 bp each, upstream and downstream).

### Ability to degrade chlorimuron-ethyl.

The abilities of the knockout strains and the complementary strains to degrade chlorimuron-ethyl were determined by monitoring cell growth and substrate concentration. These strains (inoculum size of 1%) were inoculated into 100 mL of MSM with 100 mg · L^−1^ chlorimuron-ethyl and were cultured at 28°C for 7 days. All assays had three replicates. The value of bacterial growth (OD_600_) and the concentration of chlorimuron-ethyl were detected every day.

The collected samples were centrifuged (8,000 rpm, 10 min) to remove the bacterial precipitations. Supernatants were collected and extracted by dichloromethane in the same volume 3 times. The organic phase was combined and dried with N_2_. The samples were dissolved with 10 mL of methanol and filtered through 0.22 μm nylon filters. The concentration of chlorimuron-ethyl was determined by HPLC equipped with a Zorbax C-18 ODS Spherex column (4.6 × 250 mm, 5 μm, Agilent Technologies, Palo Alto, CA, USA). The mobile phase was 0.5% methanoic acid:methanol (30:70, vol/vol) at a flow rate of 1 mL · min^−1^, and chlorimuron-ethyl was detected at 254 nm (6, 9).

### Protein structure.

The nucleotide sequences of genes *gst*, *pnbA*, and *sulE* of strain CHL1 have been submitted to the GenBank database with accession numbers OK050582, OK050584, and OK050587, respectively. The primary, secondary, and tertiary structures of the enzymes encoded by the predicted functional genes *sulE* (encoding an esterase [SulE]), *pnbA* (encoding a carboxylic ester hydrolase [PnbA]), and *gst* (encoding a GST) were analyzed using DNAMAN, NCBI, and SWISS-MODEL software, respectively (36, 40, 41). The binding site of the enzymes and chlorimuron-ethyl were analyzed by AutoDock Vina software and were visualized by PyMOL (42).

### Gene expression in E. coli and enzyme purification.

The GST-encoding gene fragment was ligated into expression vector pET-28a (+) using the *Nde*I and *Hind*III restriction sites, and the constructed vector was transduced into E. coli BL21 (DE3). The expression of GST was induced with 1 mM isopropyl β-D-1-thiogalactopyranoside for 24 h at 30°C, and GST was purified by Ni-NTA agarose chromatography (43). The purity and concentration of GST were analyzed by 12% SDS-PAGE and the Bradford method (44, 45), respectively. The expression and purification of SulE and PnbA were the same as for GST, except the *Xho*I restriction site was used instead of the *Hind*III site for the expression of PnbA. The primers used in these experiments are listed in Table S3.

### Enzyme assay and biochemical characterization.

The initial concentrations of GST and chlorimuron-ethyl in 10 mL of PBS were 0.5 μg · mL^−1^ and 20 μg · mL^−1^, respectively. The enzymatic reactions were performed at 25°C for 10 min and were terminated by the addition of dichloromethane. One unit of enzyme activity of GST to chlorimuron-ethyl was defined as the amount of enzyme that converted 1 μmol of chlorimuron-ethyl into its parent acid form in 1 min under the detected condition. The optimal pH and optimal temperature of GST were determined by assaying enzyme activity in PBS buffer at ranges of pH values (4.0 to 9.0) and temperatures (20°C to 70°C), respectively. All assays were done in triplicate, and the highest enzyme activity was set to 100%. The enzyme assay and the biochemical characterization of SulE and PnbA followed the same protocol as the above method.

### Enzymatic degradation mechanisms of chlorimuron-ethyl.

Purified SulE, GST, and PnbA (10 U) were added to 10 mL of phosphate-buffered saline containing 20 mg · L^−1^ chlorimuron-ethyl and were incubated in their optimum reaction pH and temperature conditions for 24 h. The samples were measured by UPLC-Q Exactive HRMS and analyzed using the Compound Discoverer 3.2 software (46).

### Statistical analysis.

Differences were tested for statistical significance via independent-sample *t*-tests that were performed using SPSS 23.0. A *P* value of <0.05 was considered to be indicative of a statistically significant difference.

### Data availability.

The whole-genome sequence of Chenggangzhangella methanolivorans strain CHL1 was deposited into NCBI under the accession number CP081869.

## References

[1] Alla M, Badawi AM, Hassan NM, El-Bastawisy ZM, Badran EG. 2008. Effect of metribuzin, butachlor and chlorimuron-ethyl on amino acid and protein formation in wheat and maize seedlings. Pestic Biochemistry Physiol 90:8–18. doi:10.1016/j.pestbp.2007.07.003.

[2] Brown HM. 1990. Mode of action, crop selectivity, and soil relations of the sulfonylurea herbicides. Pestic Sci 29:263–281. doi:10.1002/ps.2780290304.

[3] Wang M, Zhou Q. 2005. Single and joint toxicity of chlorimuron-ethyl, cadmium, and copper acting on wheat *Triticum aestivum*. Ecotoxicol Environ Saf 60:169–175. doi:10.1016/j.ecoenv.2003.12.012. 15546632

[4] Zawoznik MS, Tomaro ML. 2005. Effect of chlorimuron-ethyl on *Bradyrhizobium japonicum* and its symbiosis with soybean. Pest Manag Sci 61:1003–1008. doi:10.1002/ps.1077. 15920784

[5] Tan H, Xu M, Li X, Zhang H, Zhang C. 2013. Effects of chlorimuron-ethyl application with or without urea fertilization on soil ammonia-oxidizing bacteria and archaea. J Hazard Mater 260:368–374. doi:10.1016/j.jhazmat.2013.05.043. 23792929

[6] Ma JP, Wang Z, Lu P, Wang HJ, Waseem S, Li SP, Huang X. 2009. Biodegradation of the sulfonylurea herbicide chlorimuron-ethyl by the strain *Pseudomonas sp.* LW3. FEMS Microbiol Lett 296:203–209. doi:10.1111/j.1574-6968.2009.01638.x. 19459953

[7] Xiong M, Hu Z, Ying Z, Cheng X, Li C. 2013. Survival of GFP-tagged *Rhodococcus sp.* D310-1 in chlorimuron-ethyl-contaminated soil and its effects on the indigenous microbial community. J Hazard Mater 252–253:347–354. doi:10.1016/j.jhazmat.2013.02.054. 23542325

[8] Yang L, Li X, Li X, Su Z, Zhang C, Zhang H. 2014. Bioremediation of chlorimuron-ethyl-contaminated soil by *Hansschlegelia sp.* strain CHL1 and the changes of indigenous microbial population and N-cycling function genes during the bioremediation process. J Hazard Mater 274:314–321. doi:10.1016/j.jhazmat.2014.04.011. 24794985

[9] Zhang X, Zhang H, Li X, Su Z, Wang J, Zhang C. 2009. Isolation and characterization of *Sporobolomyces sp.* LF1 capable of degrading chlorimuron-ethyl. J Environ Sci (China) 21:1253–1260. doi:10.1016/S1001-0742(08)62412-2. 19999974

[10] Zang H, Yu Q, Lv T, Cheng Y, Feng L, Cheng X, Li C. 2016. Insights into the degradation of chlorimuron-ethyl by *Stenotrophomonas maltophilia* D310-3. Chemosphere 144:176–184. doi:10.1016/j.chemosphere.2015.08.073. 26363318

[11] Pan X, Wang S, Shi N, Fang H, Yu Y. 2018. Biodegradation and detoxification of chlorimuron-ethyl by *Enterobacter ludwigii sp.* CE-1. Ecotoxicol Environ Saf 150:34–39. doi:10.1016/j.ecoenv.2017.12.023. 29268112

[12] Cheng Y, Zang H, Wang H, Li D, Li C. 2018. Global transcriptomic analysis of *Rhodococcus erythropolis* D310-1 in responding to chlorimuron-ethyl. Ecotoxicol Environ Saf 157:111–120. doi:10.1016/j.ecoenv.2018.03.074. 29614448

[13] Omer CA, Lenstra R, Litle PJ, Dean C, Tepperman JM, Leto KJ, Romesser JA, O'Keefe DP. 1990. Genes for two herbicide-inducible cytochromes P-450 from *Streptomyces griseolus*. J Bacteriol 172:3335–3345. doi:10.1128/jb.172.6.3335-3345.1990. 2345149 PMC209144

[14] Hussain HA, Ward JM. 2003. Enhanced heterologous expression of two Streptomyces griseolus cytochrome P450s and Streptomyces coelicolor ferredoxin reductase as potentially efficient hydroxylation catalysts. Appl Environ Microbiol 69:373–382. doi:10.1128/AEM.69.1.373-382.2003. 12514018 PMC152428

[15] Song J. 2013. Isolation and identification of nicosulfuron-degradtive fungus (Talaromyces flavus) and study of degradation mechanism. Chinese Academy of Agricultural Sciences.

[16] Zhou S. 2015. Isolation, classification, and degradation characterisics study of nicosulfuron-degrading strain. Huazhong Agricultural University.

[17] Hang B-J, Hong Q, Xie X-T, Huang X, Wang C-H, He J, Li S-P. 2012. SulE, a sulfonylurea herbicide de-esterification esterase from *Hansschlegelia zhihuaiae* S113. Appl Environ Microbiol 78:1962–1968. doi:10.1128/AEM.07440-11. 22247165 PMC3298168

[18] Zang H, Wang H, Miao L, Cheng Y, Zhang Y, Liu Y, Sun S, Wang Y, Li C. 2020. Carboxylesterase, a de-esterification enzyme, catalyzes the degradation of chlorimuron-ethyl in *Rhodococcus erythropolis* D310-1. J Hazard Mater 387:121684. doi:10.1016/j.jhazmat.2019.121684. 31784128

[19] Zhang S, Zhang C, Sun F, Zhang Z, Zhang X, Pan H, Sun P, Zhang H. 2020. Glutathione-S-transferase (GST) catalyzes the degradation of chlorimuron-ethyl by *Klebsiella jilinsis* 2N3. Sci Total Environ 729:139075. doi:10.1016/j.scitotenv.2020.139075. 32388135

[20] Zhang C, Hao Q, Zhang S, Zhang Z, Zhang X, Sun P, Pan H, Zhang H, Sun F. 2019. Transcriptomic analysis of chlorimuron-ethyl degrading bacterial strain *Klebsiella jilinsis* 2N3. Ecotoxicol Environ Saf 183:109581. doi:10.1016/j.ecoenv.2019.109581. 31446172

[21] Yang L, Li X, Li X, Su Z, Zhang C, Zhang H. 2018. Catabolic profiles dynamics during the bioremediation process of chlorimuron-ethyl contaminated soil by *Methanolivorans* CHL1^T^. Ecotoxicol Environ Saf 155:43–49. doi:10.1016/j.ecoenv.2018.02.004. 29500939

[22] Wen Y, Huang X, Zhou Y, Hong Q, Li S. 2011. *Hansschlegelia zhihuaiae sp.* nov., isolated from a polluted farmland soil. Int J Syst Evol Microbiol 61:1114–1117. doi:10.1099/ijs.0.021543-0. 20543155

[23] Xie X. 2012. Separation, purification and enzymatic properties of a carboxyleaterase responsible for the hydrolysis of metsulfuron-methyl in strain Hansschlegelia zhihuaiae S113. Nanjing Agricultural University.

[24] Thomason L, Court DL, Bubunenko M, Costantino N, Wilson H, Datta S, Oppenheim A. 2007. Recombineering: genetic engineering in bacteria using homologous recombination. Curr Protoc Mol Biol Chap 1:Unit 1.16. 10.1002/0471142727.mb0116s78 18265390

[25] Datsenko KA, Wanner BL. 2000. One-step inactivation of chromosomal genes in *Escherichia coli* K-12 using PCR products. Proc Natl Acad Sci USA 97:6640–6645. doi:10.1073/pnas.120163297. 10829079 PMC18686

[26] Yang L, Li X, Li X, Su Z, Zhang C, Xu M, Zhang H. 2015. Improved stability and enhanced efficiency to degrade chlorimuron-ethyl by the entrapment of esterase SulE in cross-linked poly (γ-glutamic acid)/gelatin hydrogel. J Hazard Mater 287:287–295. doi:10.1016/j.jhazmat.2015.01.056. 25661176

[27] Yu Z, Zhang H, Fu X, Li X, Guo Q, Yang T, Li X. 2020. Immobilization of esterase SulE in cross-linked gelatin/chitosan and its application in remediating soils polluted with tribenuron-methyl and metsulfuron-methyl. Process Biochem 98:217–223. doi:10.1016/j.procbio.2020.08.014.

[28] Yang L, Liu L, Salam N, Xiao M, Kim C, Hozzein WN, Park D, Li W, Zhang H. 2016. Chenggangzhangella methanolivorans gen. Nov., sp. nov., a member of the family Methylocystaceae, transfer of Methylopila helvetica Doronina et al. 2000 to Albibacter helveticus comb. nov. and emended description of the genus Albibacter. Int J Syst Evol Microbiol 66:2825–2830. doi:10.1099/ijsem.0.001062. 27046027

[29] Zhu S, Qiu J, Wang H, Wang X, Jin W, Zhang Y, Zhang C, Hu G, He J, Hong Q. 2018. Cloning and expression of the carbaryl hydrolase gene mcba and the identification of a key amino acid necessary for carbaryl hydrolysis. J Hazard Mater 344:1126–1135. doi:10.1016/j.jhazmat.2017.12.006. 30216972

[30] Pandey G, Dorrian SJ, Russell RJ, Brearley C, Kotsonis S, Oakeshott JG. 2010. Cloning and biochemical characterization of a novel carbendazim (methyl-1H-benzimidazol-2-ylcarbamate)-hydrolyzing esterase from the newly isolated *Nocardioides sp.* Strain SG-4G and its potential for use in enzymatic bioremediation. Appl Environ Microbiol 76:2940–2945. doi:10.1128/AEM.02990-09. 20228105 PMC2863442

[31] Bers K, Leroy B, Breugelmans P, Albers P, Lavigne R, Sørensen SR, Aamand J, De Mot R, Wattiez R, Springael D. 2011. A novel linuron hydrolase identified by genomic-proteomic analysis of phenylurea herbicide mineralization by *Variovorax sp.* strain SRS16. Appl Environ Microbiol 77:8754–8764. doi:10.1128/AEM.06162-11. 22003008 PMC3233098

[32] Shen W, Chen H, Jia K, Ni J, Yan X, Li S. 2012. Cloning and characterization of a novel amidase from *Paracoccus sp.* M-1, showing aryl acylamidase and acyl transferase activities. Appl Microbiol Biotechnol 94:1007–1018. doi:10.1007/s00253-011-3704-6. 22101784

[33] Rhoads A, Au KF. 2015. PacBio sequencing and its applications. Genomics Proteomics Bioinformatics 13:278–289. doi:10.1016/j.gpb.2015.08.002. 26542840 PMC4678779

[34] Philip N, Rodrigues KF, William T, John DV. 2016. Whole genome sequencing of *Mycobacterium tuberculosis* SB24 isolated from Sabah, Malaysia. Genom Data 9:137–139. doi:10.1016/j.gdata.2016.08.007. 27556011 PMC4987507

[35] Jiang L, Lim CJ, Jeong JC, Kim CY, Kim DH, Kim SW, Lee J. 2019. Whole-genome sequence data and analysis of *Saccharibacillus sp.* ATSA2 isolated from Kimchi cabbage seeds. Data Brief 26:104465. doi:10.1016/j.dib.2019.104465. 31534997 PMC6743023

[36] Liu Y, Zou Z, Hu Z, Wang W, Xiong J. 2019. Morphology and molecular analysis of *Moesziomyces antarcticus* isolated from the blood samples of a Chinese patient. Front Microbiol 10:1–5. 30828326 10.3389/fmicb.2019.00254 PMC6384246

[37] Poteete AR. 2001. What makes the bacteriophage λ red system useful for genetic engineering: molecular mechanism and biological function. FEMS Microbiol Lett 201:9–14. doi:10.1111/j.1574-6968.2001.tb10725.x. 11445160

[38] Zhang C, Hao Q, Zhang Z, Zhang X, Pan H, Zhang J, Zhang H, Sun F. 2019. Whole genome sequencing and analysis of chlorimuron-ethyl degrading bacteria *Klebsiella pneumoniae* 2N3. Int J Mol Sci 20:3053. doi:10.3390/ijms20123053. 31234527 PMC6627577

[39] Yamamoto S, Izumiya H, Morita M, Arakawa E, Watanabe H. 2009. Application of λ Red recombination system to Vibrio cholerae genetics: simple methods for inactivation and modification of chromosomal genes. Gene 438:57–64. doi:10.1016/j.gene.2009.02.015. 19268696

[40] Zhan Y, Guo S. 2015. Three-dimensional (3D) structure prediction and function analysis of the chitin-binding domain 3 protein HD73-3189 from *Bacillus thuringiensis* HD73. BME 26:S2019–S2024. doi:10.3233/BME-151506. 26405978

[41] Lu S, Wang J, Chitsaz F, Derbyshire MK, Geer RC, Gonzales NR, Gwadz M, Hurwitz DI, Marchler GH, Song JS, Thanki N, Yamashita RA, Yang M, Zhang D, Zheng C, Lanczycki CJ, Marchler-Bauer A. 2020. CDD/SPARCLE: the conserved domain database in 2020. Nucleic Acids Res 48:D265–D268. doi:10.1093/nar/gkz991. 31777944 PMC6943070

[42] Forli S, Huey R, Pique ME, Sanner MF, Goodsell DS, Olson AJ. 2016. Computational protein–ligand docking and virtual drug screening with the AutoDock suite. Nat Protoc 11:905–919. doi:10.1038/nprot.2016.051. 27077332 PMC4868550

[43] Min J, Chen W, Hu X. 2019. Biodegradation of 2,6-dibromo-4-nitrophenol by *Cupriavidus sp.* strain CNP-8: kinetics, pathway, genetic and biochemical characterization. J Hazard Mater 361:10–18. doi:10.1016/j.jhazmat.2018.08.063. 30176407

[44] Laemmli UK. 1970. Cleavage of structural proteins during the assembly of the head of bacteriophage T4. Nature 227:680–685. doi:10.1038/227680a0. 5432063

[45] Badford MM. 1976. A rapid and sensitive method for the quantitation of microgram quantities of protein utilizing the principle of protein-dye binding. Anal Biochem 72:248–254. doi:10.1016/0003-2697(76)90527-3. 942051

[46] Zhao Y, Bai XL, Liu YM, Liao X. 2021. Determination of fipronil and its metabolites in egg samples by UHPLC coupled with Q-Exactive high resolution mass spectrometry after magnetic solid-phase extraction. Microchem J 169:106540. doi:10.1016/j.microc.2021.106540.

