# Supplementary material for: Whole-Genome Sequencing of a Chlorimuron-Ethyl-Degrading Strain: Chenggangzhangella methanolivorans CHL1 and Its Degrading Enzymes
Source: Microbiol Spectr. 2022 Jul 21;10(4):e01822-22. doi: 10.1128/spectrum.01822-22 (PMC9430300; doi:10.1128/spectrum.01822-22)
Supplement: Supplemental file 1 — Supplemental material. Download spectrum.01822-22-s0001.pdf, PDF file, 0.5 MB [file spectrum.01822-22-s0001.pdf]

Supplementary Material

FIGURES

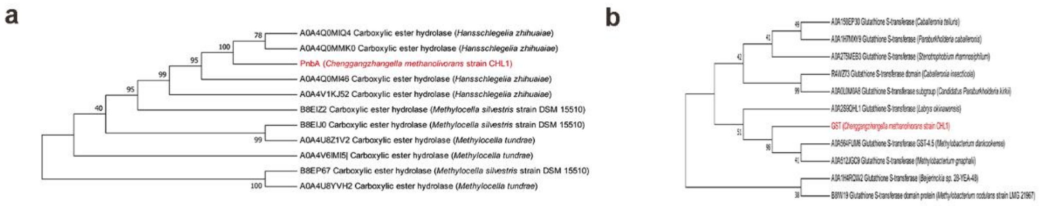

FIG S1. Neighbor-joining phylogenetic tree constructed of PnbA and GST. The bootstrap values were shown at the branch points (expressed as percentages of 1000 replications).

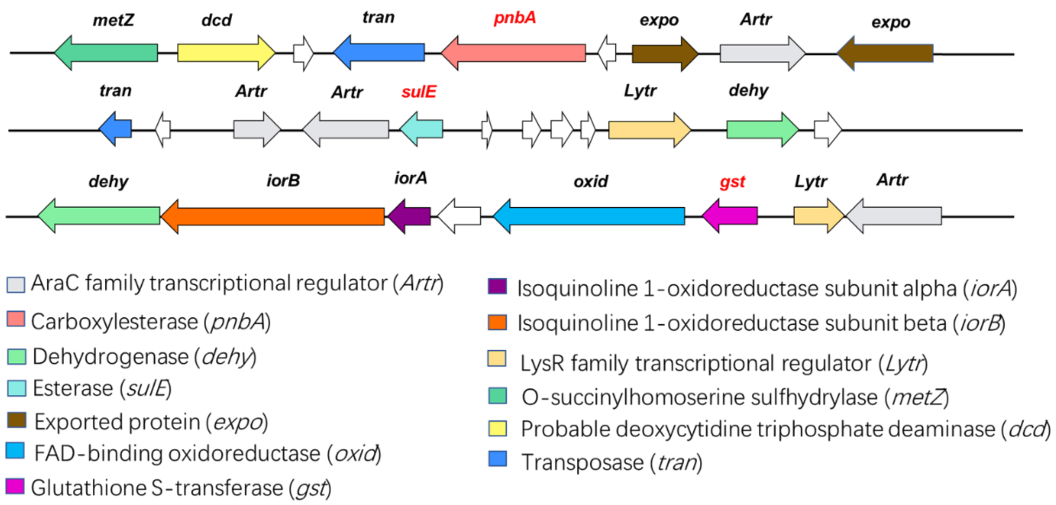

FIG S2. Gene clusters of *sulE*, *pnbA* and *gst* in the genome of strain CHL1.

a **b**

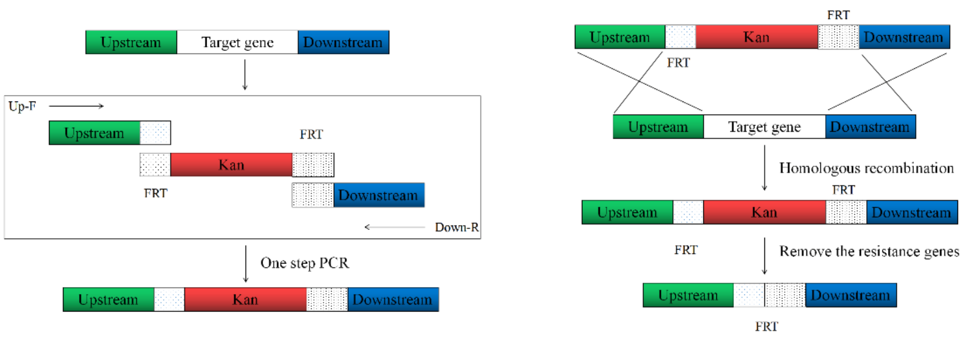

FIG S3. The schematic diagram of gene knockout in strains. (a) one-step PCR construction of a knockout linear box (b) homologous recombination.

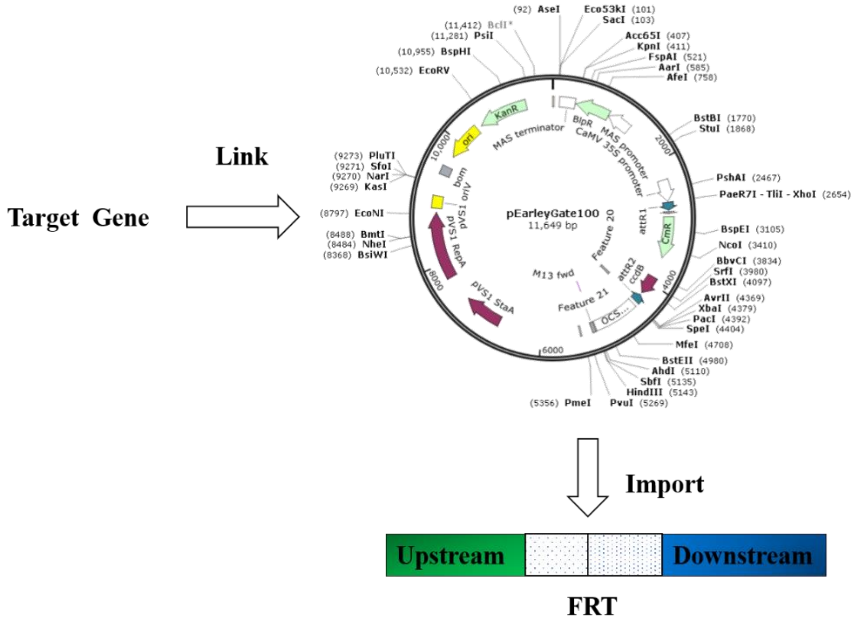

FIG S4. The schematic diagram of gene completation of knockout strains.

TABLES

TABLE S1. Basic information of chlorimuron-ethyl degradation key genes

Gene name	Annotation	Locus_tag (NCBI)	KEGG	Swissprot
sulE	Putative hydrolase	K6K41_02220	_	_
gst	Glutathione S-transferase	K6K41_17220	Drug metabolism-cytochrome P450 and Metabolism of xenobiotics by cytochrome P450	Protein GstA
pnbA	Carboxylesterase	K6K41_05595	Para-nitrobenzyl esterase	Pyrethroid hydrolase

TABLE S2. The primer sequences used in gene knockout and gene complementation

Primers	Sequences (5'-3')
FKF-*sulE*-F	TAGTAACTCACATAGGTCCTTGTGTTCTGGTGACCCATTCGGCTTCCGGAGT ACTGGGCTGTGTAGGCTGGAGCTG
FKF-*sulE*-R	CCCTCCACTCGTTAAAGTGGAGGGATGATGTCAGAATGCAGCCTCTAGGAC TTGCGGCTATCCTCCTTAGTTCCTATTCC
FKF-*pnbA*-F	ATAAGGCCGCCGGCCGGCCCGGCGTCACCGCCGCGCCAAAGCAGCTTTAG GGAGGCTGAGTGTGTAGGCTGGAGCTG
FKF-*pnbA*-R	ACCCGCGCCAACCATGCGACCGCTGCCCAAATCGCAGCAGCCGCTAACCCT ATTCGGACATCCTCCTTAGTTCCTATTCC
FKF-*gst*-F	CATCTTCGTACCGTCAGGCGGACGATGGTCCGTCGGAAACTGCCGAGGGA AGGCAAAGCCTGTGTAGGCTGGAGCTG
FKF-*gst*-R	GGCTTTGATCGCCGCCGCCGTCATGCCCGAGACAGGCTCGGGCACGACGA GGAGACCCCATCCTCCTTAGTTCCTATTCC
gst(XH)-F	GCTCTAGAATGAAGCTCCACCACCATCCGC
gst(XH)-R	CCCAAGCTTTCAGGCCGCAAGGCCCAC
pnbA(XS)-F	GCTCTAGAATGACTGGAAGGATTGGAACGTG
pnbA(XS)-R	GGACTAGTCTAAGCGATAATTGCTTGGCAGGAG
sulE(XH)-F	GCTCTAGAATGCGAGTCGCGACACAC
sulE(XH)-R	CCCAAGCTTTCAGCTTTCGTTCTGATCTAAGCCG
sulE-F	ATGCGAGTCGCGACACACG
sulE-R	TCAGCTTTCGTTCTGATCTAAGCC
pnbA-F	ATGACTGGAAGGATTGGAACGTG
pnbA-R	CTAAGCGATAATTGCTTGGCAGGA
gst-F	ATGAAGCTCCACCACCATCCG
gst-R	TCAGGCCGCAAGGCCCAC
Q-*sulE*-F	AGGGGCAATATTCCTACCTT
Q-*sulE*-R	CCGTCGACAAATGTATACTGATCA
Q-*pnbA*-F	ACGACAAGCGTCGCGACG
Q-*pnbA*-R	GCTGCTTCTCCAGGTCTTCG
Q-*gst*-F	AGACGTGTGGTGCGCGTC
Q-*gst*-R	GCGTCGCCCAGACGTCGC

"FKF-X" represents the primers of gene knockout. "X(XH)"and "X(XS)" represent the primers of gene complementation. "X" and "Q-X" represent the primers of validation of mutants. "XH" represents the restriction sites of Xba I and Hind III, and "XS" represents the restriction sites of Xba I and Spe I. "X" represent internal primer (target gene) and "Q-X" represent external primer target gene with 300bp each upstream and downstream.

TABLE S3. The primer sequences used in enzyme expression of this study

Primers	Sequences (5'-3')
sulE(NH)-F	CTGATTGCATATGGAAACTGACAACGTGGAGCT
sulE(NH)-R	TACAAGCTTGCTTTCGTTCTGATCTAAGC
gst(NH)-F	TTCCATATGATGAAGCTCCACCACCATCCG
gst(NH)-R	CCCAAGCTTTCAGGCCGCAAGGCCCA
pnbA(NX)-F	TTCCATATGATGACTGGAAGGATTGGAACGTG
pnbA(NX)-R	CCCTCGAGCTAAGCGATAATTGCTTGGCAGG

"X(NH)" and "X(NX)" represent the primers of enzyme expression. "NH" represents the restriction sites of Nde I and Hind III, and "NX" represents the restriction sites of Nde I and Xho I.
